# Crystal structure of 4-methyl-*N*-{[1-(4-methyl­benzo­yl)piperidin-4-yl]meth­yl}benzamide

**DOI:** 10.1107/S1600536814021965

**Published:** 2014-10-15

**Authors:** K. Prathebha, D. Reuben Jonathan, S. Sathya, R. Vasanthi, G. Usha

**Affiliations:** a PG and Research Department of Physics, Queen Mary’s College, Chennai-4, Tamilnadu, India; b Department of Chemistry, Madras Christian College, Chennai-59, India

**Keywords:** crystal structure, benzamide, piperdine derivatives, biological activity, hydrogen bonding

## Abstract

In the title compound, C_22_H_27_N_2_O_2_, the piperidine ring adopts a half-chair conformation with the benzene rings inclined in a *trans* orientation with respect to the piperidine ring [dihedral angle between the benzene rings = 89.1 (1)°]. In the crystal, a three-centre asymmetric N—H⋯O/C—H⋯O hydrogen-bonding inter­action leads to the formation of chains extending along the *a-*axis direction.

## Related literature   

For the synthesis of the title compound, see: Prathebha *et al.* (2013 ▶, 2014 ▶). For the biological activity of piperdine derivatives, see: Prostakov & Gaivoronskaya (1978 ▶); O’Hagan (2000 ▶); Pinder (1992 ▶). For related structures, see: Prathebha *et al.* (2014 ▶); Luo *et al.* (2011 ▶).
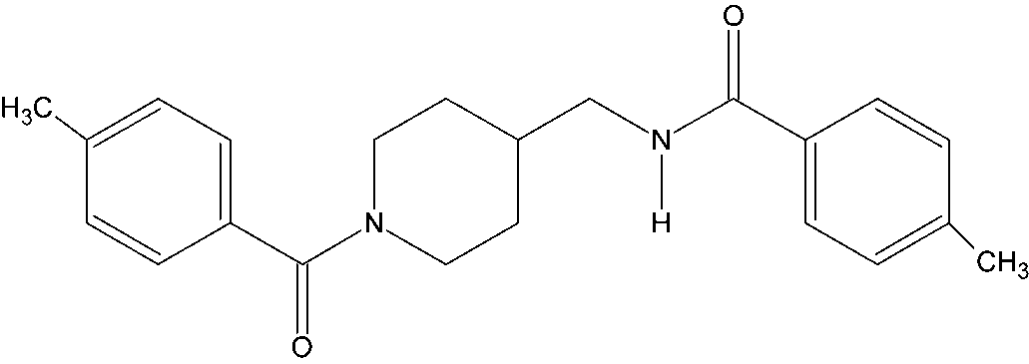


## Experimental   

### Crystal data  

C_22_H_26_N_2_O_2_
*M* *_r_* = 350.45
Orthorhombic, 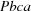

*a* = 15.3749 (6) Å
*b* = 13.1575 (5) Å
*c* = 19.2929 (9) Å
*V* = 3902.9 (3) Å^3^
*Z* = 8
Mo *K*α radiation
μ = 0.08 mm^−1^
*T* = 293 K
0.20 × 0.15 × 0.10 mm

### Data collection  

Bruker Kappa APEXII CCD diffractometer
Absorption correction: multi-scan (*SADABS*; Bruker, 2004 ▶) *T*_min_ = 0.975, *T*_max_ = 0.998
26551 measured reflections
4831 independent reflections
1847 reflections with *I* > 2σ(*I*)
*R*_int_ = 0.051

### Refinement  

*R*[*F* ^2^ > 2σ(*F* ^2^)] = 0.060
*wR*(*F* ^2^) = 0.172
*S* = 0.93
4831 reflections
235 parameters
H-atom parameters constrained
Δρ_max_ = 0.18 e Å^−3^
Δρ_min_ = −0.22 e Å^−3^

### 

Data collection: *APEX2* (Bruker, 2004 ▶); cell refinement: *APEX2* and *SAINT* (Bruker, 2004 ▶); data reduction: *SAINT* and *XPREP* (Bruker, 2004 ▶); program(s) used to solve structure: *SHELXS97* (Sheldrick, 2008 ▶); program(s) used to refine structure: *SHELXL97* (Sheldrick, 2008 ▶); molecular graphics: *ORTEP-3 for Windows* (Farrugia, 2012 ▶); software used to prepare material for publication: *SHELXL97*.

## Supplementary Material

Crystal structure: contains datablock(s) I, New_Global_Publ_Block. DOI: 10.1107/S1600536814021965/zs2314sup1.cif

Structure factors: contains datablock(s) I. DOI: 10.1107/S1600536814021965/zs2314Isup2.hkl

Supporting information file. DOI: 10.1107/S1600536814021965/zs2314Isup3.cml

**Fig. 1.**
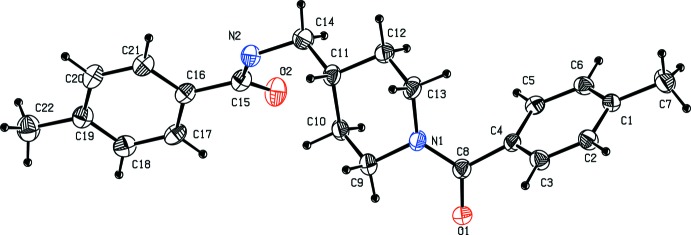
The molecular structure of the title compound showing atom numbering, with displacement ellipsoids drawn at the 30% probability level.

. DOI: 10.1107/S1600536814021965/zs2314fig1.tif
The mol­ecular structure of the title compound showing atom numbering, with displacement ellipsoids drawn at the 30% probability level.

**Fig. 2.**
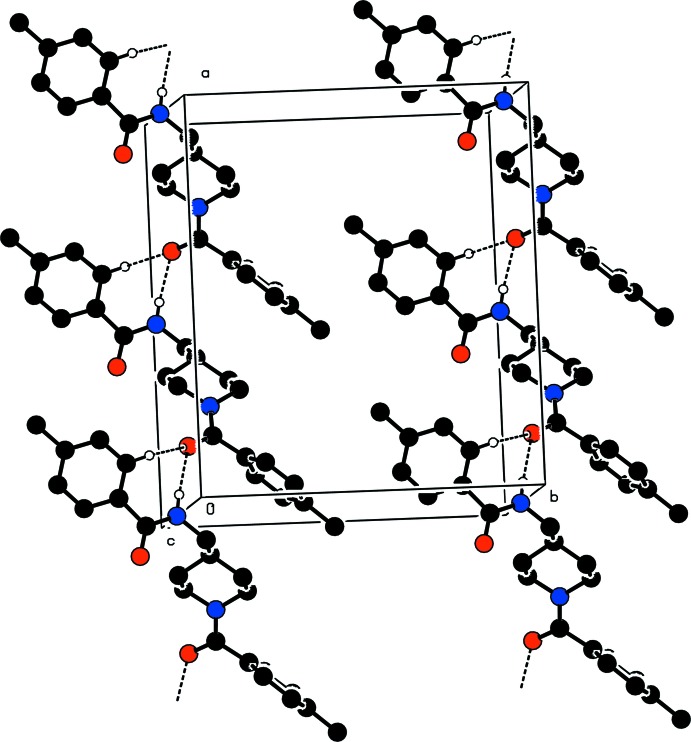
The packing of the molecules in the crystal structure. Unassociated H-atoms are omitted and dashed lines indicate the hydrogen bonds.

. DOI: 10.1107/S1600536814021965/zs2314fig2.tif
The packing of the mol­ecules in the crystal structure. Unassociated H-atoms are omitted and dashed lines indicate the hydrogen bonds.

**Fig. 3.**
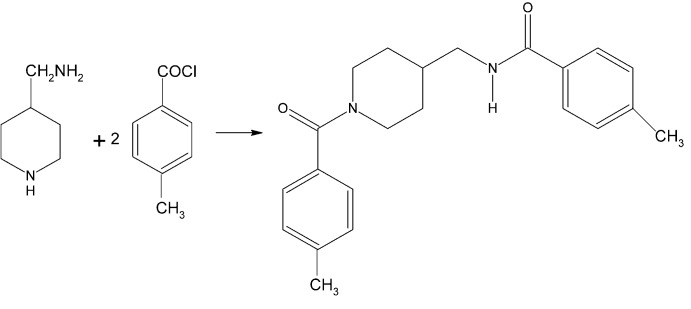
Experimental procedure

. DOI: 10.1107/S1600536814021965/zs2314fig3.tif
Experimental procedure

CCDC reference: 1027556

Additional supporting information:  crystallographic information; 3D view; checkCIF report

## Figures and Tables

**Table 1:** Hydrogen-bond geometry (, )

*D*H*A*	*D*H	H*A*	*D* *A*	*D*H*A*
N2H2*A*O1^i^	0.86	2.10	2.953(3)	169
C21H21O1^i^	0.93	2.59	3.262(3)	130

Symmetry code: (i) 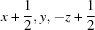.

## supplementary crystallographic information

### S1. Comment

Piperidines are an important group of compounds in the field of medicinal chemistry owing to the fact that they can frequently be found in the structures of numerous naturally occurring alkaloid and synthetic compounds with interesting biological and pharmacological properties (Prostakov *et al.*, 1978). Piperidine and its derivatives have a high impact on the medical field due to their wide range of pharmacological activities. The piperidine ring system is a ubiquitous structural component of naturally occurring alkaloid and pharmaceuticals (O'Hagan *et al.*, 2000; Pinder *et al.*, 1992). We report in this communication, the synthesis and crystal structure of a new piperidine derivative, the title compound C_22_H_27_N_2_O_2_.

In the title compound (Fig. 1), the bond lengths in the substituted benzene rings *A* and *B* are in good agreement with literature values. The C—N distances [1.460 (3)–1.523 (3) Å] are in the normal range and are in good agreement with those in similar reported structures (Prathebha *et al.*, 2013; Luo *et al.*, 2011). The bond angles around the N1 and N2 atoms [359.6 (1)° and 360.0 (2)°, respectively], show *sp*^3^ hybridization of the atoms. The two benzene rings *A* and *B* (C1–C6 and C16–C21) are inclined to one another [dihedral angle = 89.1 (1)°]. The piperdine ring (C9/C10/C11/C12/C13/N1) adopts a half-chair conformation with puckering parameters of q2 = 0.0280 (2) Å, φ2 = 114.04(5.18)°, q3 = 0.5563 (2) Å, QT = 0.5570 (2) Å and θ2 = 2.89 (2)°.

In the crystal, the molecules are linked by a asymmetric three-centre cyclic hydrogen-bonding interaction involving N1—H and C21—H donors and O1^i^ (Table 1), giving an *R*^1^_2_(7) motif and forming a chain which extends along the *a* axis (Fig. 2).

### S2. Experimental

The procedure (Prathebha *et al.*, 2013; 2014) adopted in the synthesis of a typical diamide is re-presented here (Fig. 3). 4-Aminomethylpiperidine (0.03 mol) was placed in a 250 mL round-bottomed flask and 120 mL of ethyl methyl ketone was added and the mixture was stirred at room temperature. After 10 minutes, triethylamine (0.06 mol) was added and the mixture was stirred for a further 15 minutes. 4-Methylbenzoyl chloride (0.06 mol) was then added and the reaction mixture was stirred at room temperature for about 3 h. A white precipitate of triethylammonium chloride was generated which was filtered and the filtrate was evaporated to obtain the crude product which was recrystallized twice from ethyl methyl ketone, giving the title compound: yield: 79%.

### S3. Refinement

H atoms were positioned geometrically and treated as riding on their parent atoms with C—H = 0.93–0.98 Å and N—H = 0.86 Å, with *U*_iso_(H) = 1.5*U*_eq_(C-methyl) or 1.2*U*_eq_(N, C) for other H atoms.

### Crystal data

**Table:**

C_22_H_26_N_2_O_2_	*F*(000) = 1504
*M**_r_* = 350.45	*D*_x_ = 1.193 Mg m^−^^3^
Orthorhombic, *P**b**c**a*	Mo *K*α radiation, λ = 0.71073 Å
Hall symbol: -P 2ac 2ab	Cell parameters from 4831 reflections
*a* = 15.3749 (6) Å	θ = 2.3–28.3°
*b* = 13.1575 (5) Å	µ = 0.08 mm^−^^1^
*c* = 19.2929 (9) Å	*T* = 293 K
*V* = 3902.9 (3) Å^3^	Block, colourless
*Z* = 8	0.20 × 0.15 × 0.10 mm

### Data collection

**Table:**

Bruker Kappa APEXII CCD diffractometer	4831 independent reflections
Radiation source: fine-focus sealed tube	1847 reflections with *I* > 2σ(*I*)
Graphite monochromator	*R*_int_ = 0.051
ω and φ scan	θ_max_ = 28.3°, θ_min_ = 2.3°
Absorption correction: multi-scan (*SADABS*; Bruker, 2004)	*h* = −17→20
*T*_min_ = 0.975, *T*_max_ = 0.998	*k* = −17→12
26551 measured reflections	*l* = −12→25

### Refinement

**Table:**

Refinement on *F*^2^	Primary atom site location: structure-invariant direct methods
Least-squares matrix: full	Secondary atom site location: difference Fourier map
*R*[*F*^2^ > 2σ(*F*^2^)] = 0.060	Hydrogen site location: inferred from neighbouring sites
*wR*(*F*^2^) = 0.172	H-atom parameters constrained
*S* = 0.93	*w* = 1/[σ^2^(*F*_o_^2^) + (0.0588*P*)^2^ + 1.589*P*] where *P* = (*F*_o_^2^ + 2*F*_c_^2^)/3
4831 reflections	(Δ/σ)_max_ < 0.001
235 parameters	Δρ_max_ = 0.18 e Å^−^^3^
0 restraints	Δρ_min_ = −0.22 e Å^−^^3^

### Special details

**Table:**

Geometry. All e.s.d.'s (except the e.s.d. in the dihedral angle between two l.s. planes) are estimated using the full covariance matrix. The cell e.s.d.'s are taken into account individually in the estimation of e.s.d.'s in distances, angles and torsion angles; correlations between e.s.d.'s in cell parameters are only used when they are defined by crystal symmetry. An approximate (isotropic) treatment of cell e.s.d.'s is used for estimating e.s.d.'s involving l.s. planes.
Refinement. Refinement of *F*^2^ against ALL reflections. The weighted *R*-factor *wR* and goodness of fit *S* are based on *F*^2^, conventional *R*-factors *R* are based on *F*, with *F* set to zero for negative *F*^2^. The threshold expression of *F*^2^ > σ(*F*^2^) is used only for calculating *R*-factors(gt) *etc*. and is not relevant to the choice of reflections for refinement. *R*-factors based on *F*^2^ are statistically about twice as large as those based on *F*, and *R*- factors based on ALL data will be even larger.

### Fractional atomic coordinates and isotropic or equivalent isotropic displacement parameters (Å^2^)

**Table:**

	*x*	*y*	*z*	*U*_iso_*/*U*_eq_	
C22	0.6965 (2)	−0.4676 (2)	0.47887 (18)	0.0948 (10)	
H22A	0.7516	−0.4377	0.4902	0.114*	
H22B	0.7033	−0.5122	0.4399	0.114*	
H22C	0.6753	−0.5055	0.5179	0.114*	
N1	0.24058 (14)	0.06231 (14)	0.21465 (13)	0.0678 (7)	
C1	−0.00836 (15)	0.31788 (17)	0.17840 (14)	0.0539 (7)	
C2	0.03275 (17)	0.28524 (18)	0.11916 (15)	0.0634 (7)	
H2	0.0216	0.3183	0.0775	0.076*	
C3	0.08988 (16)	0.20487 (19)	0.12001 (15)	0.0631 (7)	
H3	0.1158	0.1835	0.0790	0.076*	
C4	0.10897 (14)	0.15576 (17)	0.18134 (14)	0.0504 (6)	
C5	0.06856 (16)	0.18834 (19)	0.24129 (14)	0.0566 (7)	
H5	0.0809	0.1563	0.2831	0.068*	
C6	0.01028 (15)	0.26771 (18)	0.23982 (14)	0.0571 (7)	
H6	−0.0170	0.2880	0.2806	0.069*	
C7	−0.07222 (18)	0.4045 (2)	0.17671 (17)	0.0831 (9)	
H7A	−0.0943	0.4163	0.2225	0.100*	
H7B	−0.1195	0.3878	0.1462	0.100*	
H7C	−0.0436	0.4647	0.1603	0.100*	
C8	0.16482 (16)	0.0623 (2)	0.18032 (15)	0.0582 (7)	
C9	0.29448 (18)	−0.0291 (2)	0.21712 (17)	0.0756 (9)	
H9A	0.3451	−0.0199	0.1878	0.091*	
H9B	0.2616	−0.0865	0.1995	0.091*	
C10	0.32335 (16)	−0.05074 (18)	0.29030 (15)	0.0653 (8)	
H10A	0.3618	−0.1092	0.2904	0.078*	
H10B	0.2730	−0.0672	0.3184	0.078*	
C11	0.37020 (15)	0.03968 (18)	0.32161 (15)	0.0595 (7)	
H11	0.4215	0.0535	0.2930	0.071*	
C12	0.31090 (17)	0.13214 (18)	0.31724 (16)	0.0704 (8)	
H12A	0.2602	0.1211	0.3463	0.084*	
H12B	0.3415	0.1912	0.3348	0.084*	
C13	0.28185 (17)	0.15248 (19)	0.24409 (17)	0.0758 (9)	
H13A	0.2410	0.2087	0.2437	0.091*	
H13B	0.3316	0.1714	0.2160	0.091*	
C14	0.40150 (18)	0.0214 (2)	0.39565 (15)	0.0701 (8)	
H14A	0.4287	0.0831	0.4126	0.084*	
H14B	0.3514	0.0076	0.4247	0.084*	
C15	0.43907 (18)	−0.1546 (2)	0.42462 (14)	0.0650 (7)	
C16	0.50989 (17)	−0.2317 (2)	0.43383 (13)	0.0596 (7)	
C17	0.48785 (18)	−0.3334 (2)	0.42988 (14)	0.0682 (8)	
H17	0.4311	−0.3516	0.4186	0.082*	
C18	0.5486 (2)	−0.4082 (2)	0.44237 (15)	0.0724 (8)	
H18	0.5324	−0.4760	0.4381	0.087*	
C19	0.63257 (19)	−0.3850 (2)	0.46102 (14)	0.0695 (8)	
C20	0.65476 (19)	−0.2835 (2)	0.46459 (16)	0.0776 (9)	
H20	0.7112	−0.2656	0.4770	0.093*	
C21	0.59503 (18)	−0.2076 (2)	0.45019 (14)	0.0688 (8)	
H21	0.6123	−0.1399	0.4515	0.083*	
N2	0.46281 (13)	−0.06195 (16)	0.40288 (12)	0.0649 (6)	
H2A	0.5165	−0.0514	0.3928	0.078*	
O1	0.13858 (11)	−0.01377 (14)	0.14932 (11)	0.0784 (6)	
O2	0.36348 (13)	−0.17709 (15)	0.43739 (12)	0.0903 (7)	

### Atomic displacement parameters (Å^2^)

**Table:**

	*U*^11^	*U*^22^	*U*^33^	*U*^12^	*U*^13^	*U*^23^
C22	0.094 (2)	0.103 (2)	0.088 (3)	0.014 (2)	−0.0037 (19)	0.0161 (19)
N1	0.0524 (13)	0.0512 (13)	0.0998 (19)	0.0038 (11)	−0.0208 (13)	−0.0054 (12)
C1	0.0500 (15)	0.0491 (14)	0.0626 (19)	0.0003 (12)	−0.0012 (13)	−0.0051 (13)
C2	0.0760 (19)	0.0608 (16)	0.0534 (18)	0.0120 (15)	−0.0029 (14)	0.0044 (13)
C3	0.0696 (18)	0.0681 (17)	0.0518 (18)	0.0097 (15)	0.0032 (13)	−0.0024 (14)
C4	0.0442 (14)	0.0494 (14)	0.0575 (17)	−0.0011 (11)	−0.0046 (12)	−0.0013 (13)
C5	0.0592 (17)	0.0620 (16)	0.0486 (17)	−0.0039 (14)	−0.0040 (13)	0.0010 (13)
C6	0.0566 (16)	0.0605 (16)	0.0543 (18)	−0.0043 (14)	0.0087 (13)	−0.0079 (13)
C7	0.079 (2)	0.0688 (18)	0.102 (3)	0.0132 (16)	0.0057 (17)	−0.0044 (17)
C8	0.0517 (16)	0.0571 (17)	0.0659 (19)	−0.0026 (13)	−0.0026 (14)	−0.0014 (14)
C9	0.0630 (18)	0.0685 (18)	0.095 (3)	0.0176 (15)	−0.0140 (16)	−0.0107 (17)
C10	0.0535 (16)	0.0542 (16)	0.088 (2)	0.0099 (13)	−0.0041 (15)	−0.0025 (15)
C11	0.0425 (14)	0.0619 (16)	0.074 (2)	−0.0021 (13)	0.0008 (13)	0.0039 (14)
C12	0.0559 (16)	0.0542 (16)	0.101 (3)	−0.0060 (13)	−0.0106 (16)	−0.0082 (15)
C13	0.0550 (17)	0.0574 (17)	0.115 (3)	−0.0053 (14)	−0.0228 (16)	0.0050 (17)
C14	0.0614 (17)	0.0717 (18)	0.077 (2)	−0.0026 (15)	0.0065 (15)	−0.0039 (15)
C15	0.0598 (19)	0.080 (2)	0.0554 (18)	−0.0082 (16)	0.0033 (14)	0.0081 (15)
C16	0.0611 (18)	0.0724 (19)	0.0451 (16)	−0.0070 (15)	−0.0011 (12)	0.0101 (13)
C17	0.0668 (18)	0.082 (2)	0.0558 (19)	−0.0141 (17)	−0.0077 (14)	0.0083 (15)
C18	0.084 (2)	0.0709 (19)	0.062 (2)	−0.0063 (18)	−0.0051 (15)	0.0078 (15)
C19	0.070 (2)	0.083 (2)	0.0557 (19)	0.0009 (17)	0.0004 (14)	0.0113 (15)
C20	0.0625 (19)	0.091 (2)	0.079 (2)	−0.0083 (18)	−0.0071 (15)	0.0125 (18)
C21	0.0663 (19)	0.0714 (18)	0.069 (2)	−0.0080 (16)	−0.0049 (15)	0.0095 (15)
N2	0.0518 (13)	0.0738 (15)	0.0691 (17)	−0.0011 (12)	0.0025 (11)	0.0105 (12)
O1	0.0663 (12)	0.0677 (12)	0.1013 (17)	0.0074 (10)	−0.0196 (11)	−0.0242 (11)
O2	0.0584 (13)	0.1027 (15)	0.1098 (18)	−0.0099 (11)	0.0146 (11)	0.0269 (13)

### Geometric parameters (Å, º)

**Table:**

C22—C19	1.505 (4)	C10—H10A	0.9700
C22—H22A	0.9600	C10—H10B	0.9700
C22—H22B	0.9600	C11—C12	1.523 (3)
C22—H22C	0.9600	C11—C14	1.526 (4)
N1—C8	1.340 (3)	C11—H11	0.9800
N1—C13	1.460 (3)	C12—C13	1.504 (4)
N1—C9	1.461 (3)	C12—H12A	0.9700
C1—C2	1.375 (3)	C12—H12B	0.9700
C1—C6	1.386 (3)	C13—H13A	0.9700
C1—C7	1.505 (3)	C13—H13B	0.9700
C2—C3	1.375 (3)	C14—N2	1.452 (3)
C2—H2	0.9300	C14—H14A	0.9700
C3—C4	1.380 (3)	C14—H14B	0.9700
C3—H3	0.9300	C15—O2	1.224 (3)
C4—C5	1.381 (3)	C15—N2	1.340 (3)
C4—C8	1.500 (3)	C15—C16	1.499 (4)
C5—C6	1.376 (3)	C16—C17	1.382 (3)
C5—H5	0.9300	C16—C21	1.383 (3)
C6—H6	0.9300	C17—C18	1.379 (4)
C7—H7A	0.9600	C17—H17	0.9300
C7—H7B	0.9600	C18—C19	1.374 (4)
C7—H7C	0.9600	C18—H18	0.9300
C8—O1	1.233 (3)	C19—C20	1.381 (4)
C9—C10	1.507 (4)	C20—C21	1.384 (4)
C9—H9A	0.9700	C20—H20	0.9300
C9—H9B	0.9700	C21—H21	0.9300
C10—C11	1.516 (3)	N2—H2A	0.8600
			
C19—C22—H22A	109.5	C10—C11—C12	108.7 (2)
C19—C22—H22B	109.5	C10—C11—C14	113.5 (2)
H22A—C22—H22B	109.5	C12—C11—C14	111.5 (2)
C19—C22—H22C	109.5	C10—C11—H11	107.6
H22A—C22—H22C	109.5	C12—C11—H11	107.6
H22B—C22—H22C	109.5	C14—C11—H11	107.6
C8—N1—C13	124.8 (2)	C13—C12—C11	111.8 (2)
C8—N1—C9	120.6 (2)	C13—C12—H12A	109.2
C13—N1—C9	114.2 (2)	C11—C12—H12A	109.2
C2—C1—C6	117.8 (2)	C13—C12—H12B	109.2
C2—C1—C7	121.2 (2)	C11—C12—H12B	109.2
C6—C1—C7	120.9 (2)	H12A—C12—H12B	107.9
C1—C2—C3	121.6 (2)	N1—C13—C12	110.4 (2)
C1—C2—H2	119.2	N1—C13—H13A	109.6
C3—C2—H2	119.2	C12—C13—H13A	109.6
C2—C3—C4	120.4 (2)	N1—C13—H13B	109.6
C2—C3—H3	119.8	C12—C13—H13B	109.6
C4—C3—H3	119.8	H13A—C13—H13B	108.1
C3—C4—C5	118.5 (2)	N2—C14—C11	114.4 (2)
C3—C4—C8	119.6 (2)	N2—C14—H14A	108.7
C5—C4—C8	121.5 (2)	C11—C14—H14A	108.7
C6—C5—C4	120.7 (2)	N2—C14—H14B	108.7
C6—C5—H5	119.6	C11—C14—H14B	108.7
C4—C5—H5	119.6	H14A—C14—H14B	107.6
C5—C6—C1	120.9 (2)	O2—C15—N2	122.8 (3)
C5—C6—H6	119.6	O2—C15—C16	120.2 (3)
C1—C6—H6	119.6	N2—C15—C16	117.1 (2)
C1—C7—H7A	109.5	C17—C16—C21	117.8 (3)
C1—C7—H7B	109.5	C17—C16—C15	118.1 (2)
H7A—C7—H7B	109.5	C21—C16—C15	124.0 (3)
C1—C7—H7C	109.5	C16—C17—C18	121.0 (3)
H7A—C7—H7C	109.5	C16—C17—H17	119.5
H7B—C7—H7C	109.5	C18—C17—H17	119.5
O1—C8—N1	121.6 (2)	C19—C18—C17	121.6 (3)
O1—C8—C4	119.0 (2)	C19—C18—H18	119.2
N1—C8—C4	119.4 (2)	C17—C18—H18	119.2
N1—C9—C10	110.7 (2)	C18—C19—C20	117.4 (3)
N1—C9—H9A	109.5	C18—C19—C22	120.9 (3)
C10—C9—H9A	109.5	C20—C19—C22	121.7 (3)
N1—C9—H9B	109.5	C19—C20—C21	121.6 (3)
C10—C9—H9B	109.5	C19—C20—H20	119.2
H9A—C9—H9B	108.1	C21—C20—H20	119.2
C9—C10—C11	111.4 (2)	C16—C21—C20	120.5 (3)
C9—C10—H10A	109.4	C16—C21—H21	119.7
C11—C10—H10A	109.4	C20—C21—H21	119.7
C9—C10—H10B	109.4	C15—N2—C14	122.7 (2)
C11—C10—H10B	109.4	C15—N2—H2A	118.7
H10A—C10—H10B	108.0	C14—N2—H2A	118.7
			
C6—C1—C2—C3	0.7 (4)	C14—C11—C12—C13	178.4 (2)
C7—C1—C2—C3	−178.7 (2)	C8—N1—C13—C12	132.7 (3)
C1—C2—C3—C4	−1.4 (4)	C9—N1—C13—C12	−55.1 (3)
C2—C3—C4—C5	0.8 (4)	C11—C12—C13—N1	55.1 (3)
C2—C3—C4—C8	174.3 (2)	C10—C11—C14—N2	60.6 (3)
C3—C4—C5—C6	0.3 (3)	C12—C11—C14—N2	−176.3 (2)
C8—C4—C5—C6	−173.0 (2)	O2—C15—C16—C17	25.0 (4)
C4—C5—C6—C1	−1.0 (3)	N2—C15—C16—C17	−155.7 (2)
C2—C1—C6—C5	0.5 (3)	O2—C15—C16—C21	−151.0 (3)
C7—C1—C6—C5	179.9 (2)	N2—C15—C16—C21	28.4 (4)
C13—N1—C8—O1	170.9 (3)	C21—C16—C17—C18	0.5 (4)
C9—N1—C8—O1	−0.8 (4)	C15—C16—C17—C18	−175.7 (2)
C13—N1—C8—C4	−11.1 (4)	C16—C17—C18—C19	1.8 (4)
C9—N1—C8—C4	177.3 (2)	C17—C18—C19—C20	−2.1 (4)
C3—C4—C8—O1	−63.0 (3)	C17—C18—C19—C22	176.1 (3)
C5—C4—C8—O1	110.2 (3)	C18—C19—C20—C21	0.2 (4)
C3—C4—C8—N1	118.9 (3)	C22—C19—C20—C21	−178.0 (3)
C5—C4—C8—N1	−67.9 (3)	C17—C16—C21—C20	−2.4 (4)
C8—N1—C9—C10	−132.0 (3)	C15—C16—C21—C20	173.6 (3)
C13—N1—C9—C10	55.5 (3)	C19—C20—C21—C16	2.1 (4)
N1—C9—C10—C11	−55.6 (3)	O2—C15—N2—C14	2.9 (4)
C9—C10—C11—C12	55.8 (3)	C16—C15—N2—C14	−176.4 (2)
C9—C10—C11—C14	−179.5 (2)	C11—C14—N2—C15	−99.6 (3)
C10—C11—C12—C13	−55.8 (3)		

### Hydrogen-bond geometry (Å, º)

**Table:**

*D*—H···*A*	*D*—H	H···*A*	*D*···*A*	*D*—H···*A*
N2—H2*A*···O1^i^	0.86	2.10	2.953 (3)	169
C21—H21···O1^i^	0.93	2.59	3.262 (3)	130
C9—H9*B*···O1	0.97	2.33	2.738 (3)	104
C14—H14*B*···O2	0.97	2.45	2.794 (3)	100

Symmetry code: (i) *x*+1/2, *y*, −*z*+1/2.
