# Supplementary figures and images for: Crystal structure of 4-methyl-N-{[1-(4-methyl­benzo­yl)piperidin-4-yl]meth­yl}benzamide
Source: Acta Crystallogr Sect E Struct Rep Online. 2014 Oct 15;70(Pt 11):o1157. doi: 10.1107/S1600536814021965 (PMC4257237; doi:10.1107/S1600536814021965)

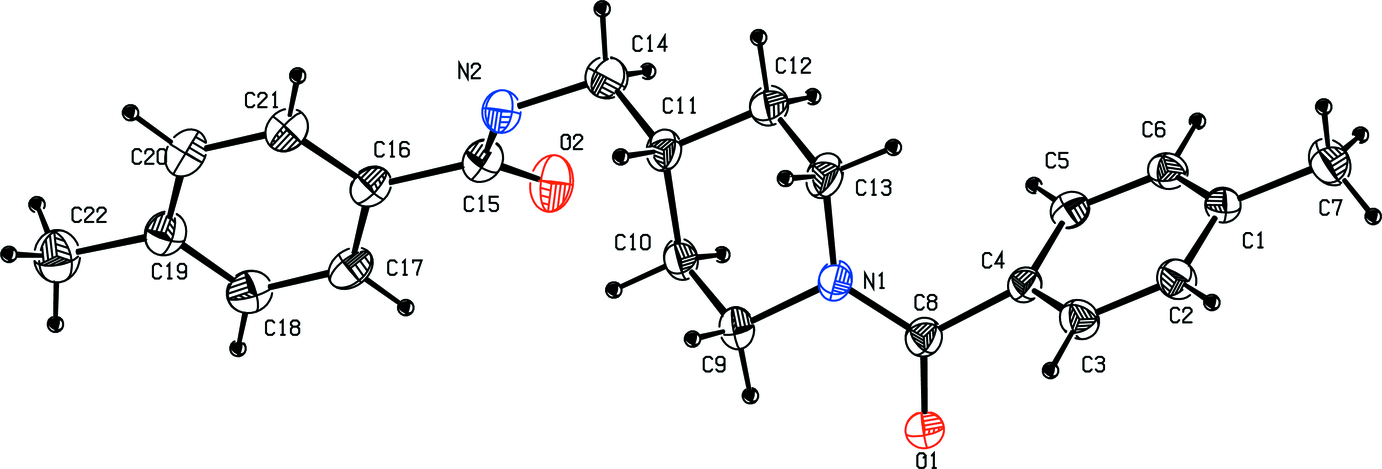

Supplement: Supplementary file 4 [file e-70-o1157-fig1.tif]

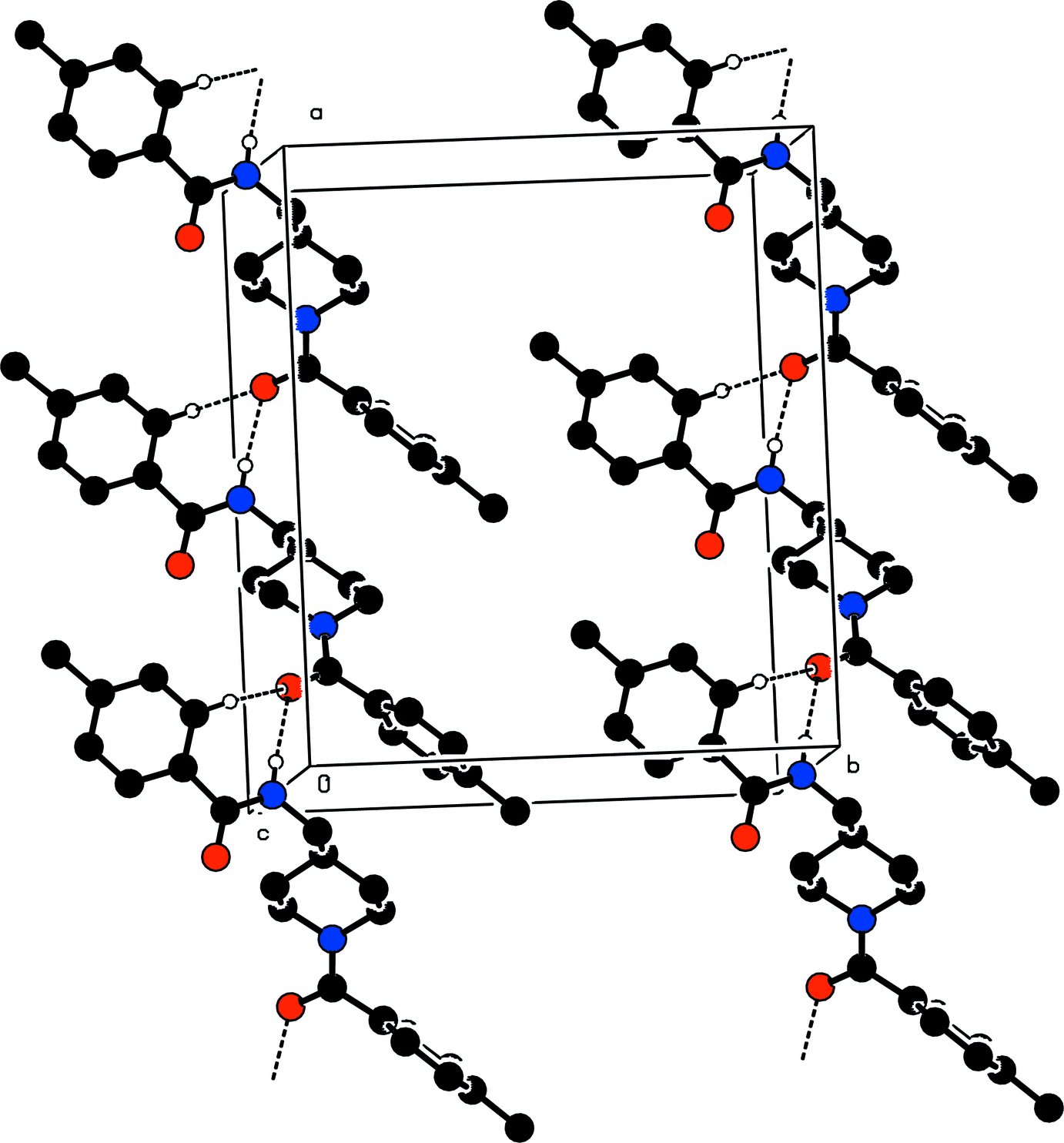

Supplement: Supplementary file 5 [file e-70-o1157-fig2.tif]
